# Tremor-Dominant Movement Disorder in *ANKRD11-* Associated KBG Syndrome

**DOI:** 10.5334/tohm.926

**Published:** 2024-09-25

**Authors:** Antonia M. Stehr, Thomas Koeglsperger, Maureen Jacob, Valerio Rhodio, Juliane Winkelmann, Franziska Hopfner, Michael Zech

**Affiliations:** 1 Institute of Human Genetics, Technical University of Munich, School of Medicine and Health, Munich, Germany; 2 Department of Neurology, LMU University Hospital, LMU Munich, Munich, Germany; 3 Department of Translational Brain Research, German Centre for Neurodegenerative Diseases, Munich, Germany; 4 Institute of Neurogenomics, Helmholtz Munich, Neuherberg, Germany; 5 DZPG, Deutsches Zentrum für Psychische Gesundheit, Munich, Germany; 6 Munich Cluster for Systems Neurology (SyNergy), Munich, Germany; 7 Institute for Advanced Study, Technical University of Munich, Garching, Germany

**Keywords:** KBG syndrome, *ANKRD11*, tremor, combined tremor syndrome

## Abstract

**Background:**

KBG syndrome is a monogenic disorder caused by heterozygous pathogenic variants in *ANKRD11*. A recent single-case study suggested that the clinical spectrum of KBG syndrome, classically defined by distinctive craniofacial traits and developmental delay, may include movement disorders.

**Case report:**

We report a 24-year-old patient harboring a pathogenic *de novo ANKRD11* frameshift variant. The phenotype was dominated by a progressive tremor-dominant movement disorder, characterized by rest, intention and postural tremor of the hands, voice tremor, head and tongue tremor, increased muscle tone and signs of ataxia. Additionally, the patient had a history of mild developmental delay and epilepsy.

**Discussion:**

Adding to the recently described individual, our present patient highlights the relevance of movement disorders as a clinically relevant manifestation of KBG syndrome. *ANKRD11* pathogenic variants should be considered in the differential diagnosis of combined tremor syndromes.

## Introduction

KBG syndrome (MIM 148050, ORPHA 2332) is a genetic neurodevelopmental disorder characterized by developmental delay (DD), learning difficulties or intellectual disability (ID) and distinct craniofacial features [1,2]. Additional observed features are seizures, microcephaly, changes in brain anatomy, psychiatric and behavioral abnormalities, conductive hearing disorders, eye abnormalities and congenital heart defects [1,3]. KBG syndrome is caused by single nucleotide pathogenic variants and small indels in *ANKRD11* or lager copy number variants (mostly deletions) at 16q24.3 involving *ANKRD11*. Most variants are loss-of-function variants, with haploinsufficiency of *ANKRD11* considered to be the pathogenic mechanism [1]. *ANKRD11* encodes a protein that plays an important role in chromatin remodeling via histone acetylation during neuronal development and thus regulates gene expression. Definitive genotype-phenotype correlations do not exist [1].

Recently, the phenotypic spectrum of KBG syndrome was proposed to be extended by the umbrella term “epileptic dyskinetic encephalopathy” due to the description of a patient with KBG syndrome characterized by an early-onset developmental disorder with epileptic encephalopathy and dyskinetic movement disorder [4]. This patient had persistent daily seizures despite treatment with multiple antiepileptic drugs, whereas in most patients with KBG-syndrome the epilepsy responds well to antiepileptic treatment [1,4]. In addition, this was the first report of an association of variants in *ANKRD11* with hyperkinetic movements. The movement disorder was characterized as infantile-onset jittery movements of the limbs and head, progressing into a mixed hyperkinetic movement disorder including choreoathetosis. Until then, movement disorders were not considered part of the phenotypic spectrum of KBG syndrome and are largely unreported in this context [4]. We now present a second patient of an *ANKRD11*-associated movement disorder and the first patient of an *ANKRD11*-associated tremor-dominant syndrome. We describe a 24-year-old male patient with a *de novo* frameshift duplication in *ANKRD11*, who displayed progressive and therapy-resistant intention, postural and resting tremor.

After review of the literature, this unusual presentation expands the phenotypic spectrum of KBG syndrome and adds *ANKRD11* variants to the list of genetic alterations associated with tremor-dominant movement disorders.

## Patient description

The boy first came to medical attention at the age of six weeks with pronounced sleep myoclonus. In addition, fluttering of the tongue when drinking and trembling of the lower lip, especially when excited, were observed. Subsequently, the infant had tremor of the hands (left>right), microcephaly and mild developmental delay, particularly in the development of motor skills with gait instability. Free walking was achieved at 18 months. The intensity of the tremor gradually increased with age. Awake-sleep EEGs performed at the age of 6 weeks and 8 weeks, as well as at 2 years after a fever-associated seizure, showed normal results without epileptic activity. An extended examination at the age of 16 years revealed epileptic activity on the EEG in the form of low- to medium-amplitude generalized, frontally accentuated (poly) spike-wave paroxysms. Follow-up EEGs continued to show the above described pathologic pattern, as a result of which antiepileptic therapy with levetiracetam (2× 1500 mg/d) was supplemented with topiramate (2× 50 mg/d) and carbamazepine (400 mg/d). An MRI examination of the head showed unremarkable findings apart from a suspected small arachnoid cyst in the left sylvian fissure and single small medullary lesions. Thyroid dysfunction or Wilson’s disease were excluded as the cause of the tremor by biochemical screening.

**Figure 1:**
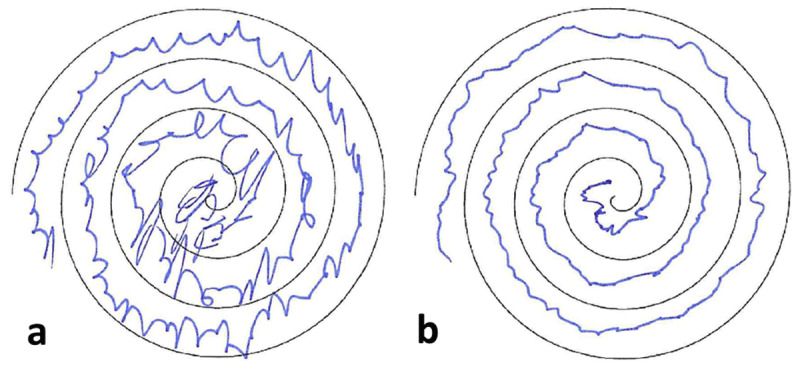
Visualization of the patient’s action tremor using Achimedean spiral. **a** left hand **b** right hand. The patient is right-handed.

Currently, at the age of 24 years, the patient presented with a rest tremor and a fine-beating postural tremor of the arms and legs, as well as an intention tremor of the hands (Figure 1 and Video 1). In addition, he exhibited a voice tremor, mild head tremor, mild tongue tremor and increased muscle tone. There were significant restrictions in daily life due to tremor-related fine motor impairment (use of cutlery, drinking, writing). A response of the tremor to alcohol was not reported. A therapeutic approach with propranolol (2 × 40 mg/d) was unsuccessful, while primidone was not tolerated due to nausea. In the course of further detailed diagnostics, finger-to-nose test and heel-to-shin test showed bilateral dysmetria and hyperreflexia and spasticity of the upper and lower extremities were observed. In addition to the tremor and epilepsy, the patient presented with a dysplastic right kidney, chronic obstipation (especially in childhood), astigmatism with strabismus and a myopia (–3 Dpt.). Due to proteinuria, therapy with candesartan was carried out, the kidney function was unimpaired. The patient showed distinctive craniofacial features (triangular face, microcephalus, thin lips, protruding ears), an abduction deficit of the third and fourth finger and clinodactyly of the fifth fingers (Video 1). The second incisors and premolars in all four quadrants were absent. Since childhood the patient experienced recurrent middle ear infections (currently 2–3 times a year), so tympanostomy tubes were inserted. Hearing was not impaired.

**Video 1:** Video documentation of the patient’s tremor-dominant movement disorder (including a jerky component) upon consultation at our institution. **Segment A** shows the patient’s resting tremor of the upper and lower extremities. **Segment B** documents the patient’s postural tremor of the hands. **Segment C** demonstrates the action tremor of the left hand. The patient showed pronounced action tremor of both hands (left>right) when tracing the Archimedean spiral. Note characteristic facial features: microcephaly, triangular face, thin lips, protruding ears.

To further evaluate an underlying cause of the patient’s symptoms, trio whole-exome sequencing was performed at the age of 24 years. The analysis revealed a *de novo* heterozygous frameshift variant (NM_001256182.2: c.5469dup, p.(Pro1824SerfsTer126)) in *ANKRD11* that was classified as pathogenic (ACMG criteria PVS1, PS2, PM2_supp and PP5). The variant predicts a frameshift and premature termination of protein translation, resulting in KBG syndrome in the patient.

## Discussion

The patient’s symptoms were overall consistent with the previously described phenotype of KBG syndrome. However, the progressive tremor-dominant movement disorder, which significantly determined the phenotype of our patient, broadens the previously known phenotypic spectrum of the condition. To date, only one case of a movement disorder in association with an *ANKRD11* variant has been described [4].

Tremor is one of the most common movement disorders in adults [5] and is defined as an “involuntary, rhythmic, oscillatory movement of a body part”. The consensus statement of the International Parkinson and Movement Disorder Society published in 2018 introduces a new biaxial tremor classification, whereby tremor is categorized according to clinical features (Axis 1) and etiology (Axis 2) [6]. Within Axis 1, a distinction is made between the presence of an “isolated” tremor (such as essential tremor, ET) or the occurrence of tremor as part of a more complex (neurological) disorder (“combined tremor”) [6,7].

Tremor is genetically heterogeneous despite high phenotypic similarity. Most monogenic tremors occur as part of an overarching syndrome and are part of a neurologic phenotypic spectrum (“combined” tremor syndromes) [6,7]. The Online Mendelian Inheritance in Man (OMIM) database lists 532 results (04/2024) when searching for the keyword “tremor”. In particular, numerous monogenic neurodegenerative, neuropathic and metabolic diseases manifest with tremor often accompanied by signs of other movement disorders, especially of a dystonic (e.g. *ANO3*, *SGCE*), ataxic (e.g. *PPPP2R2B, ATX3, TBP)* or parkinsonian (e.g. *LRRK2*, *FMR1*, *ATX3)* type. In some of these conditions, including autosomal dominant cranio-cervical dystonia (DYT-*ANO3*) and different subtypes of spinocerebellar ataxia (including SCA12 and SCA40), an initial presentation as an “isolated” tremor before an onset of further neurological symptoms in the form of dystonia or ataxia has been described, potentially leading to a misdiagnosis of ET (ET-like syndromes). This is further complicated by the fact that in many cases of ET other neurological findings occur in the course of time, which, however, in their severity do not allow for an alternative neurological diagnosis (so called soft signs). This concept of ET with other neurological signs of uncertain significance such as tandem gait impairment or mild memory impairment was recently introduced as “ET plus” [5,7].

On this basis, monogenic tremor syndromes are categorized into “isolated” tremor syndromes (ET-like syndromes), when the tremor is (yet) the only manifestation of the disease and “combined” tremor syndromes (either as “ET plus syndromes” or “tremor combined with other neurological and non-neurological features”) [7]. ET on the other hand, is defined as isolated action tremor of the upper extremity of a duration of at least three years in the absence of other neurological symptoms. Other parts of the body (lower limb, head and voice) may also be affected in the context of ET [8].

The fact that the here presented patient did not have an isolated action tremor but also a pronounced postural tremor, rest tremor and minor intention tremor in combination with accompanying spasticity, signs of ataxia and other systemic abnormalities clearly distinguishes the clinical picture from that of an ET. We therefore diagnose a monogenic combined tremor syndrome classified as “tremor combined with other neurological and non-neurological features” in our patient in the context of a KBG syndrome. Consequently, we propose *ANKRD11*-KBG syndrome to be included in the list of monogenic causes of a combined tremor syndrome. Retrospective and prospective analyses of cohorts with pathogenic *ANKRD11* variants will be crucial to confirm the occurrence of movement disorders in this condition, which will be important for guiding clinical management and identifying potential therapeutic targets based on the gene’s known function. It should be noted that although trio exome sequencing revealed no other significant findings related to the patient’s symptoms, another monogenic cause for the tremulous movement disorder, while unlikely, cannot be completely ruled out due to technical limitations of exome sequencing.
